# Effect of Exclusion Diets on Symptom Severity and the Gut Microbiota in Patients With Irritable Bowel Syndrome

**DOI:** 10.1016/j.cgh.2021.05.027

**Published:** 2021-05-20

**Authors:** Adrienne Lenhart, Tien Dong, Swapna Joshi, Nancee Jaffe, Charlene Choo, Cathy Liu, Jonathan P. Jacobs, Venu Lagishetty, Wendy Shih, Jennifer S. Labus, Arpana Gupta, Kirsten Tillisch, Emeran A. Mayer, Lin Chang

**Affiliations:** * Vatche and Tamar Manoukian Division of Digestive Diseases, Los Angeles, California; ‡ G Oppenheimer Center for Neurobiology of Stress and Resilience, Los Angeles, California; § David Geffen School of Medicine, Los Angeles, California; ¶ Semel Institute for Neuroscience and Human Behavior, University of California, Los Angeles, California; ∥ Division of Gastroenterology, Hepatology and Parenteral Nutrition, Veterans Administration Greater Los Angeles Healthcare System, Los Angeles, California

**Keywords:** Irritable Bowel Syndrome, Diet, Fecal Microbiome

## Abstract

**BACKGROUND & AIMS:**

Altered fecal microbiota have been reported in irritable bowel syndrome (IBS), although studies vary, which could be owing to dietary effects. Many IBS patients may eliminate certain foods because of their symptoms, which in turn may alter fecal microbiota diversity and composition. This study aimed to determine if dietary patterns were associated with IBS, symptoms, and fecal microbiota differences reported in IBS.

**METHODS:**

A total of 346 IBS participants and 170 healthy controls (HCs) completed a Diet Checklist reflecting the diet(s) consumed most frequently. An exclusion diet was defined as a diet that eliminated food components by choice. Within this group, a gluten-free, dairy-free, or low fermentable oligosaccharides, disaccharides, monosaccharides, and polyols diet was further defined as restrictive because they often are implicated in reducing symptoms. Stool samples were obtained from 171 IBS patients and 98 HCs for 16S ribosomal RNA gene sequencing and microbial composition analysis.

**RESULTS:**

Having IBS symptoms was associated with consuming a restrictive diet (27.17% of IBS patients vs 7.65% of HCs; odds ratio, 3.25; 95% CI, 1.66–6.75; *P* value = .006). IBS participants on an exclusion or restrictive diet reported more severe IBS symptoms (*P* = .042 and .029, respectively). The composition of the microbiota in IBS patients varied depending on the diet consumed. IBS participants on an exclusion diet had a greater abundance of *Lachnospira* and a lower abundance of *Eubacterium* (q value, <.05), and those on a restrictive diet had a lower abundance of *Lactobacillus* (q value, <.05).

**CONCLUSIONS:**

Restrictive diets likely are consumed more by IBS patients than HCs to reduce GI symptom severity. Dietary patterns influence the composition of the fecal microbiota and may explain some of the differences between IBS and HCs.

## Graphical Abstract

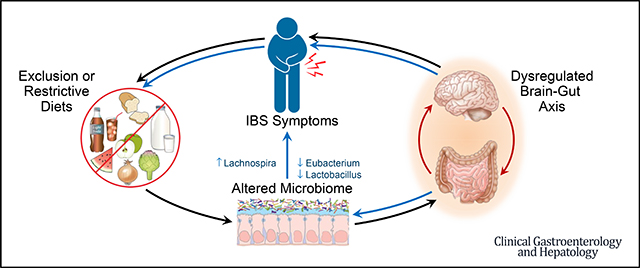

Irritable bowel syndrome (IBS) is a disorder of brain–gut interaction, defined by recurring episodes of abdominal pain and alterations in stool form and frequency.^1^ The pathogenesis of IBS likely is multifactorial and includes alterations in gut microbiota. Most patients with IBS report meal-related gastrointestinal (GI) symptoms^2^ and will eliminate foods thought to provoke symptoms.^3^ Restrictive diets used to treat IBS symptoms include gluten- or lactose-free diets, or those low in fermentable oligosaccharides, disaccharides, monosaccharides, and polyols (FODMAPs).^4–6^

Changes in diet also can influence the composition of the intestinal microbiota. Multiple studies have reported variances in gut microbiota profiles in patients with IBS compared with healthy controls (HCs), although differences have been inconsistent.^7^ In addition, previous studies largely have been underpowered, have not assessed dietary intake or antibiotic use, and often have not corrected for multiple comparisons.^7^ The relative abundance of different microbial taxa species in IBS may be altered by both restrictive diets and more general exclusion diets that eliminate certain food groups by choice, but are not necessarily implemented to alleviate GI symptoms. For instance, relative microbial abundance may be altered by diets that reduce fiber intake,^8^ eliminate animal proteins,^9^ high FODMAP foods^10,11^ or gluten,^12^ or increase plant or vegetable consumption.^9^

Although multiple studies have evaluated fecal microbiota in IBS, only a few studies have assessed the effect of diet on the microbiome in IBS.^7^ These studies have focused on the low FODMAP diet, which may be associated with a decreased abundance of fecal *Bifidobacteria* and *Clostridium* cluster XIVa and a higher abundance of *Ruminococcus*.^10,11^ However, further research is needed to determine if the broader categories of restrictive or exclusion diets contribute to differences in microbial profiles in IBS and HCs.

The present study aimed to compare dietary patterns, including the consumption of restrictive or exclusion diets, between IBS participants and HCs, and to determine if these diets were associated with IBS symptom severity and alterations in the fecal microbiota. We aimed to test the hypotheses that restrictive diets are associated with having IBS, more severe GI symptoms, and altered microbial compositions.

## Methods

### Participants

Adults with IBS and HCs were recruited to this study between July 2013 and June 2019. Most of the participants were recruited through community advertisements for clinical research studies. A lesser proportion of participants were recruited from GI clinics. This study was approved by the Institutional Review Board at the University of California, Los Angeles.

All participants underwent a medical history and physical examination. The diagnosis of IBS was made using the Rome III or IV criteria,^1,13^ depending on the year of recruitment and after the exclusion of organic disease. IBS was subclassified as constipation-predominant IBS (IBS-C), diarrhea-predominant IBS (IBS-D), IBS with mixed symptoms (IBS-M), or IBS unclassified. HCs had no history of GI symptoms or an organic GI disease. Participants who submitted stool samples for microbiota analysis were excluded if they had received antibiotics within the previous 3 months. Additional details regarding methods and inclusion and exclusion criteria can be found in the Supplementary Methods section.

### Questionnaires

#### Bowel Symptom Questionnaire.

The Bowel Symptom Questionnaire^14^ includes Rome diagnostic questions for IBS and questions that assess the severity of IBS symptoms. The severity of abdominal pain and bloating were measured using numeric scales ranging from 0 to 20 (0 indicates no pain/sensation and 20 indicates the most intense pain/sensation).

#### Irritable Bowel Syndrome Severity Scoring System.

The IBS Severity Scoring System (IBS-SSS) is a validated instrument that assesses the frequency and severity of abdominal pain, severity of abdominal distention, dissatisfaction with bowel habits, and interference of IBS with daily life over a 10-day period.^15^ Each of the 5 categories is scored from 0 to 100, and the total IBS-SSS is the sum of these categories (total score range, 0–500).

### Diet Questionnaires

The Diet Checklist is a questionnaire developed by our institution, intended to represent the diet that best reflects what individuals consume on a regular basis. These diets include a standard or modified American, Mediterranean, vegan, vegetarian, gluten-free, dairy-free, and low FODMAP diet (described in detail in Supplementary Table 1). Confirmation of a specific diet was verified with the Diet History Questionnaire-II (DHQ-II)^16^ (a food frequency questionnaire) and 24-hour diet diaries (see the Supplementary Methods section).

Diets were classified further as either standard or exclusion diets. A standard diet was defined as an American diet or a Mediterranean diet because these diets included most food groups. Exclusion diets were defined as those that eliminated certain food groups by choice, and included dairy-free, gluten-free, low FODMAP, vegan, vegetarian, and/or Paleo diets. A subcategory of an exclusion diet was a restrictive diet, defined by diets that likely were initiated to reduce GI symptoms and included gluten-free, dairy-free, and/or low FODMAP diets.

### Psychological Symptoms

GI symptom–related anxiety was measured using the Visceral Sensitivity Index (range, 0–90).^17^ Current psychological symptoms were assessed using the Hospital Anxiety and Depression Scale (HADS). Higher HADS anxiety (range, 0–21) and depression (range, 0–21) represent more severe anxiety or depression, respectively.

### 16S Ribosomal RNA Gene Sequencing and Microbial Composition Analysis

Stool samples were obtained from IBS participants and HCs. DNA was extracted from frozen fecal samples using the PowerSoil DNA Isolation Kit (MO BIO Laboratories, Carlsbad, CA) with bead beating following the manufacturer’s protocol. The V4 hypervariable region of the 16S ribosomal RNA gene was amplified using the 515F and 806R primers. Polymerase chain reaction products were purified by a commercial kit and the DNA was sequenced using an Illumina HiSeq 2500 (Illumina, San Diego, CA). The merged pair-end reads were processed using QIIME 1.9.1^18^ with default settings. Taxonomic assignments of sequences were performed using closed reference operational taxonomic unit (OTU) picking in QIIME against the Greengenes database (Second Genome, Inc, Brisbane, CA) preclustered at 97% identity. OTUs were removed if they were present in less than 15% of samples and a total of 2619 OTUs were evaluated. Sequence depths ranged from 32,306 to 676,638 per sample, with a median value of 112,598. *β*-diversity was calculated using the DEICODE plugin in QIIME 2, which uses a robust Aitchison analysis that accounts for the sparse compositional nature of microbiome data. This method has been shown to yield higher discriminatory power compared with other common metrics, such as UniFrac or Bray-Curtis.^19^

*β*-diversity was modeled for association with demographic and clinical factors using the Adonis package in R (R Core Team, 2019), which implements a permutational analysis of variances using distance matrices. *β*-diversity was visualized using principal components analysis. *α*-diversity was measured using the Shannon Index, a measurement of species evenness and richness, with data rarefied to 32,306 sequences.^20^ a-diversity was tested using analysis of variance in R. Differential abundance testing was performed using DESEq2 in R, which uses a Bayesian approach to fit nonrarified count data to a negative binomial model.^21^ *P* values were converted to q values to correct for multiple hypothesis testing and a q-value of 0.05 or less was deemed significant.^22^

### Statistical Analysis for Clinical and Diet Data

Group comparisons for demographic characteristics between IBS and HCs were performed using regression and chi-square tests. A generalized linear model (GLM) (family: binomial [link = “logit”]) was used to evaluate the dietary differences between IBS compared with HCs, while adjusting for age, sex, race, body mass index, and HADS anxiety. We calculated odds ratios (ORs), 95% CIs associated with the ORs, Z-values, and *P* values. The GLM was applied using the lm function in R to test whether IBS symptoms differed by diet while controlling for age, sex, race, and body mass index. *P* values were adjusted for the number of Diet Checklist items tested (N = 11). A GLM with IBS status as a dependent variable was used to compare DHQ-II food variables between IBS and HCs. *P* values were adjusted for the number of DHQ-II food variables (N = 31) tested. A false-discovery rate (FDR) (Benjamini-Hotchberg method^23^) less than 0.05 was considered significant. All statistical analyses including the main effect of diet accounting for potential confounding variables were performed using R version 3.6.0 (http://cran.r-project.org) and were 2-tailed.

## Results

### Participant Characteristics

There were 346 IBS participants and 170 HCs included in this study (Table 1). These groups had a similar proportion of women (72.5% and 65.3%, respectively). There was a significant difference in race (*P* < .001), including a larger percentage of Asian participants in the HC group. IBS participants had higher HADS anxiety scores than HCs (*P* < .001). IBS-D was the most common IBS subtype (40.2%), followed by IBS-C (30.1%), IBS-M (22.0%), and IBS unclassified (7.2%). The mean IBS-SSS score was in the moderate range (257.35 ± 88.07).

### Dietary Preferences

The majority of participants consumed a standard American diet (74.1% of HCs and 59.5% of IBS participants) (Table 2). There was no significant relationship between consuming a standard diet and IBS status. There was a significantly higher proportion of IBS participants on a restrictive diet compared with HCs (27.2% vs 7.7%; OR, 3.25; 95% CI, 1.66–6.75; FDR-adjusted *P* value = .006).

### Association Between Diet and Irritable Bowel Syndrome Symptoms

Compared with IBS participants who were not on a standard diet, those on a standard diet had significantly lower IBS-SSS scores (248.39 ± 83.85 vs 278.95 ± 83.79; FDR-adjusted *P* = .042). In contrast, IBS participants on an exclusion or restrictive diet reported greater IBS symptom severity based on higher IBS-SSS compared with those not on an exclusion diet (278.95 ± 83.79 vs 248.39 ± 83.85; FDR-adjusted *P* value = .042) or restrictive diet (291.59 ± 87.06 vs 249.35 ± 81.57; FDR adjusted *P* value ± .029), respectively (Table 3). The relationship between increased IBS-SSS symptom severity and diet was driven primarily by an increased number of days of abdominal pain per week (*P* < .0001) and a greater interference with quality of life (*P* < .001) (Supplementary Table 2). There were no other associations between IBS symptoms or bowel habit subtypes and types of diet (Supplementary Table 3). In addition, there were significant IBS vs HC differences in DHQ-II foods including lactose, vegetables, and fiber (Supplementary Table 4).

### Microbiota Analysis in Irritable Bowel Syndrome Participants and Healthy Controls

A total of 171 (49.4%) IBS participants and 98 (57.6%) HCs submitted stool samples for microbiota analysis and had similar characteristics to the overall group. Microbial community composition differed by race (*P* = .005), which was adjusted for in additional analyses. Analyses were performed both including and excluding the 9 IBS participants (2.6%) and 2 HCs (1.18%) on probiotics, and there was no significant effect on the overall findings. Therefore, the participants on probiotics were included in the final analysis. Differences in the fecal microbiota observed between HCs and IBS included a greater abundance of Rikenellaceae and *Parabacteroides* in IBS (q-values, <0.05) (Supplementary Table 5). When adjusting for both diet and race, only Rikenellaceae showed a greater abundance in IBS (Supplementary Table 6). There were no differences in *α*- or *β*-diversity between HCs and IBS participants overall (Supplementary Figure 1) and in those on a standard diet (Supplementary Figure 2).

### Microbiota Analysis According to Irritable Bowel Syndrome Bowel Habit Subtype

**Figure 1.**
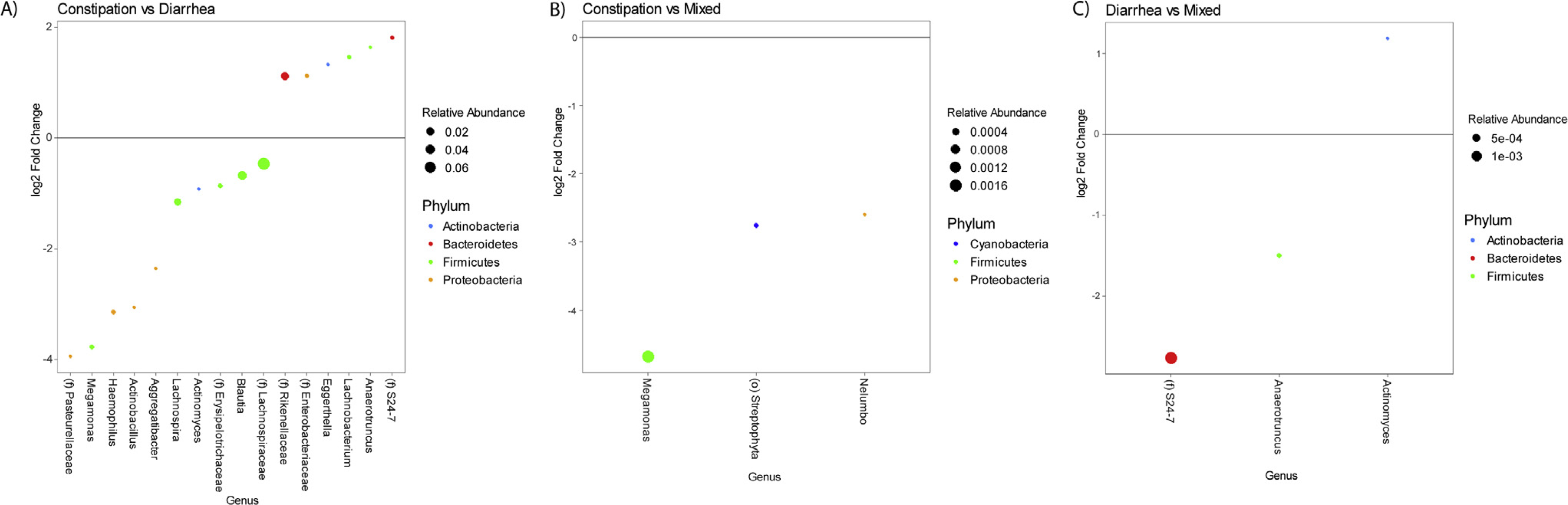
*(A)* Relative fecal abundances in constipation-predominant irritable bowel syndrome (IBS-C) compared with diarrhea-predominant irritable bowel syndrome (IBS-D). *(B)* Relative fecal abundances in IBS-C compared with IBS with mixed symptoms (IBS-M). *(C)* Relative fecal abundances in IBS-D compared with IBS-M.

A significant difference in *β*-diversity was observed between IBS bowel habit subtypes (*P* = .047). IBS-D participants had a significantly greater fecal abundance of Lachnospiraceae, *Blaudia*, *Lachnospira*, and Erysipelotrichaceae among others, and a significantly lower abundance of Rikenellaceae, *S24-7*, *Lachnobacterium*, and *Anaerotruncus* compared with IBS-C (all q-values, <0.05) (Figure 1A). IBS-C participants also had a significantly lower abundance of *Megamonas*, *Streptophyta*, and *Nelumbo* (all q-values, <0.05) compared with participants with IBS-M (Figure 1B). Finally, IBS-D participants had a significantly greater abundance of *Actinomyces* and a lower abundance of *S24-7* and *Anaerotruncus* vs IBS-M participants (Figure 1C) (all q-values, <0.05). There were no significant differences in *α*-diversity or in diet categories (standard diet, exclusion diet, restrictive diet) based on IBS bowel habit subtype.

### Diet and the Fecal Microbiota: Participants With Irritable Bowel Syndrome

Of the IBS participants who submitted stool samples, 104 were on a standard diet, 67 consumed an exclusion diet, and 39 consumed a restrictive diet. A significant difference in *β*-diversity was observed for IBS participants on a standard diet compared with those not on a standard diet (ie, on an exclusion diet) (*P* = .016). IBS participants on a standard diet showed a greater abundance of *Bifidobacterium* and *Prevotella* (all q-values, <0.05) (Supplementary Figure 3).

**Figure 2.**
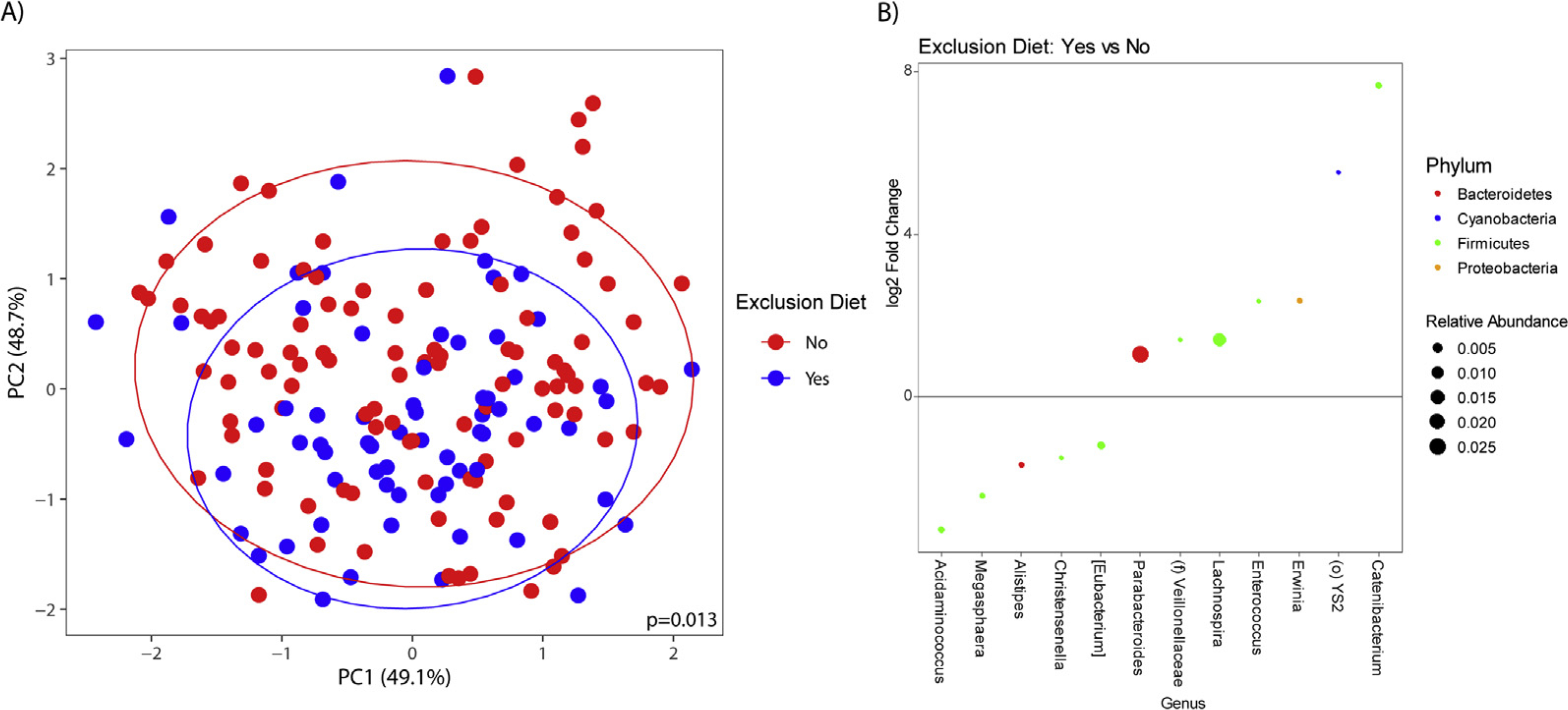
*(A) β*-diversity in irritable bowel syndrome (IBS) participants on an exclusion diet compared with IBS participants not on an exclusion diet. *(B)* Relative fecal abundances in IBS participants on an exclusion diet compared with a nonexclusion diet.

**Figure 3.**
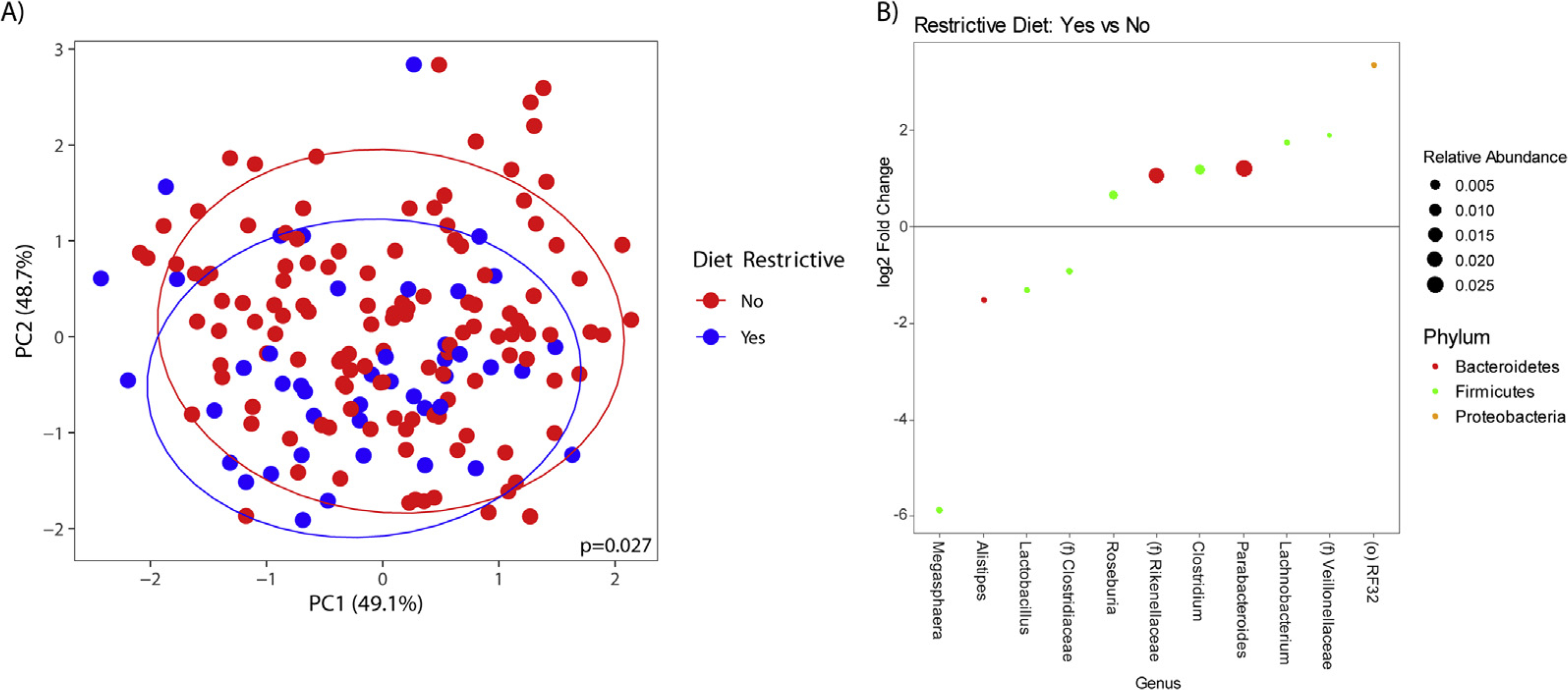
*(A) β*-diversity in irritable bowel syndrome (IBS) participants on a restrictive diet compared with IBS participants not on a restrictive diet. *(B)* Relative fecal abundances in IBS participants on a restrictive diet compared with a nonrestrictive diet.

A significant difference in *β*-diversity also was observed for IBS participants on either an exclusion diet (*P* = .013) or a restrictive diet (*P* = .027) compared with those not on an exclusion or restrictive diet, respectively. IBS participants on an exclusion diet had a significantly greater abundance of genera including *Lachnospira* (q-value, <0.05), and a lower abundance of genera such as *Eubacterium* (q-values, <0.05) (Figure 2). IBS participants who consumed a restrictive diet had a lower abundance of *Lactobacillus* (q-value, <0.05) (Figure 3). No group differences in *α*-diversity were seen.

### Diet and the Fecal Microbiota: Healthy Controls

Seventy-five HCs who submitted stool samples were on a standard diet, 17 consumed an exclusion diet, and only 7 (7.3%) were on a restrictive diet. Within HCs, there was no observed effect of diet on either *α*- or *β*-diversity. There were no significant differences in fecal bacterial abundances based on diet in the HC population.

## Discussion

Diet rarely is evaluated in studies of the microbiome and IBS. This study comprehensively assessed the relationship between exclusion and restrictive diets and the microbiome in patients with IBS. IBS participants were more likely than HCs to consume restrictive diets. Those on restrictive diets had worse IBS symptom severity, primarily driven by an increased number of days of abdominal pain and symptom-related negative impact on quality of life, and likely restricted foods to reduce IBS symptoms. Multiple studies have shown that restrictive diets, such as a low FODMAP diet^6^ and a gluten-free diet,^4,24^ can improve symptoms in IBS patients. IBS participants in our study likely initiated these types of restrictive diets to reduce GI symptoms; however, determining the effectiveness of these diets on long-term symptom reduction would require a longitudinal-based study.

Both restrictive and exclusion diets were associated with altered microbial profiles in IBS. We did not see a similar effect of these diets on the microbiome in HCs, mainly owing to HCs not having GI symptoms that warranted consuming restrictive diets. Differences in the fecal microbiota between HCs and IBS participants included a greater abundance of Rikenellaceae and *Parabacteroides* in IBS. These microbial differences between IBS participants and HCs may in part be explained by variations in diet, but also may be influenced by other disease-related, environmental, or methodologic factors. It also is possible that baseline microbial changes may have led to GI symptoms, which in turn resulted in consuming restrictive diets. However, the lack of *α*-and *β*-diversity seen between HCs and IBS participants irrespective of diet and only when evaluating those on a standard diet provides additional support that it is likely the components of exclusion and restrictive diets that were responsible for the microbial differences observed in our study.

Within IBS, a greater abundance of *Bifidobacterium* and *Prevotella* was associated with consumption of a standard diet. *Bifidobacterium* often are used as probiotics given their positive host benefits. Although current guidelines do not recommend the clinical use of probiotics in IBS,^25^ a recent systematic review and meta-analysis showed that *Bifidobacterium*-containing probiotics led to an overall reduction in IBS symptoms compared with probiotics containing *Lactobacillus* alone.^26^ IBS participants on a standard diet had lower IBS symptom severity, and thus, it is possible that the increased abundance of *Bifidobacterium* played a role in symptom reduction. The greater abundance of *Prevotella* in a standard diet can be explained by its association with diets high in complex carbohydrates and fiber.^9,27^

IBS participants on an exclusion diet had a greater abundance of genera such as *Lachnospira* compared with those not on an exclusion diet. *Lachnospira* are short-chain fatty acid producers, which can have positive effects on immune function, intestinal barrier integrity, and mucus production.^28^ Increased abundance of Lachnospira has been associated with increased vegetable intake and plant-based diets.^29^ Our definition of an exclusion diet included vegan and vegetarian diets and therefore the observed increase in *Lachnospira* aligns with previous literature. IBS participants on an exclusion diet also had a lower fecal abundance of genera such as *Eubacterium*. A decreased abundance of Eubacterium *hallii* has been associated with a gluten-free diet,^12^ which could explain our findings because a gluten-free diet was considered to be an exclusion diet.

IBS participants on a restrictive diet showed changes in fecal abundancies including a lower abundance of *Lactobacillus*, which is beneficial to human health. Reductions in *Lactobacillus* have been observed with a gluten-free diet.^30^ Because our definition of a restrictive diet included both gluten-free and dairy-free diets, the decrease in *Lactobacillus* aligns with previous literature. A summary of previous evidence pertaining to these genera can be found in Supplementary Table 7.

Our study also showed significant differences in fecal microbiota based on IBS bowel habit subtype. The most striking differences were observed when comparing participants with IBS-C with IBS-D. However, we did not observe any significant differences in diet categories based on bowel habit subtype, showing that both diet and bowel habit category affect the microbiome independently. Previous literature has been mixed regarding changes in microbial profiles according to IBS bowel habit subtype with no consistent pattern.^7^ The majority of existing studies were comprised of relatively small sample sizes and the larger sample size in our study may have allowed for the detection of microbiome differences based on bowel habit subtype.

This was a large study evaluating the microbiota in IBS patients. In addition, our study included all IBS bowel habit subtypes and controlled for diet and multiple comparisons, unlike many previous similar studies. Our study also was novel in that we evaluated the effects of restrictive and exclusion diets on IBS symptoms and fecal microbial profiles. Limitations of this study include that it was cross-sectional and participants were not randomized to receive a specific dietary intervention. Dietary pattern category was determined by each participant’s best assessment and therefore we cannot determine their dietary adherence with complete precision. However, with the guidance of our GI dietitian, we verified each exclusion diet on the Diet Checklist against their DHQ-II and 24-hour diet diaries.

In conclusion, our study showed that restrictive diets are consumed more by IBS participants than HCs, likely to reduce GI symptom severity, and these diets influence the fecal microbiota composition. Gut microbiota can induce physiologic changes in brain–gut interactions and affect IBS symptoms.^31^ Although the microbiome in patients with IBS undoubtably is variable, dietary-induced changes in the gut microbiome may explain at least some of the variability reported in the literature.

## Supplementary Material

1

## Figures and Tables

**Table 1.** Characteristics of Participants

Variable	HCs (n = 170)	IBS participants (n = 346)	Statistic^a^	*P* value
Age, y, means ± SD	28.19 ± 9.97	30.10 ± 10.84	1.94	.054
Sex, N (% female)	111 (65.3)	251 (72.5)	2.53	.112
BMI, means ± SD	24.61 ± 3.34	24.24 ± 4.49	−0.97	.336
Race, N (%)			23.23	<.0001
Caucasian	64 (40.5)	190 (59.4)		
Asian	62 (39.2)	62 (19.4)		
African American	12 (7.6)	25 (7.8)		
Other	20 (12.7)	43 (13.4)		
Education, N (%)			1.06	.787
High school graduate or less	13 (7.9)	20 (6.2)		
Some college	61 (37.2)	134 (41.2)		
College graduate	45 (27.4)	85 (26.5)		
Any postgraduate work	45 (27.4)	85 (26.2)		
Medications, N (%)				
Antidepressants	4 (2.4)	5(1.5)		
Benzodiazepines	0 (0.0)	3 (0.9)		
Antispasmodics	0 (0.0)	4(1.2)		
NSAIDs as needed	38 (22.4)	30 (8.7)		
Laxatives	0 (0.0)	6(1.7)		
Antidiarrheal agents	0 (0.0)	5(1.5)		
Fiber supplements	1 (0.6)	5(1.5)		
Probiotics	2(1.2)	9 (2.6)		
HADS anxiety (0–21), means ± SD	4.52 ± 3.36	8.51 ± 4.32	10.54	<.0001
HADS depression (0–21), means ± SD	3.68 ± 3.18	1.77 ± 1.80	7.25	<.0001
VSI score (0–90), means ± SD	4.52 ± 8.63	41.34 ± 16.33	23.87	<.0001
IBS subtype				
IBS-C		104 (30.1%)		
IBS-D		139 (40.2%)		
IBS-M		76 (22.0%)		
IBS-U		25 (7.2%)		
IBS symptoms				
Abdominal pain (0–20), means ± SD		9.21 ± 4.18		
Bloating (0–20), means ± SD		11.27 ± 5.00		
IBS-SSS^b^ (0–500), means ± SD		257.35 ± 88.07		

BMI, body mass index; HADS, Hospital Anxiety and Depression Scale; HC, healthy control; IBS-C, constipation-predominant IBS; IBS-D, diarrhea-predominant IBS; IBS-M, IBS with mixed bowel habits; IBS-SSS, Irritable Bowel Syndrome Severity Scoring System; IBS-U, IBS unclassified; NSAID, nonsteroidal antiinflammatory drug; SD, standard deviation; VSI, Visceral Sensitivity Index.

a The t value is shown for continuous variables and the chi-squared value is shown for discrete variables.

b Obtained in 336 IBS participants.

**Table 2.** Primary Dietary Patterns in IBS Participants and Healthy Controls

Diet	HCs (n = 170)	IBS participants (n = 346)	Z-value	*P* value	Adjusted *P* value (FDR)^a^
Standard diet	126 (74.1%)	206 (59.5%)	−1.22	.002	.272
American^b^	117 (68.8%)	216 (62.4%)	0.70	.184	.485
Mediterranean	26 (15.3%)	41 (11.9%)	−2.25	.346	.050
Exclusion diet	43 (25.3%)	140 (40.5%)	1.29	.001	.246
Vegan	4 (2.4%)	9 (2.6%)	−1.81	1.000	.118
Vegetarian (all subtypes)	23 (13.5%)	43 (12.4%)	−1.67	.841	.134
Paleo	7 (4.1%)	27 (7.8%)	1.16	.160	.274
Low FODMAP	2 (1.2%)	18 (5.2%)	2.36	.012	.050
Gluten-free	5 (2.9%)	52 (15.0%)	2.30	<.0001	.050
Dairy-free	6 (3.5%)	63 (18.2%)	3.26	<.0001	**.005**
Restrictive diet	13 (7.7%)	94 (27.2%)	3.31	<.0001	**.005**
Low FODMAP	2 (1.2%)	18 (5.2%)	2.36	.012	.050
Gluten-free	5 (2.9%)	52 (15.0%)	2.30	<.0001	.050
Dairy-free	6 (3.5%)	63 (18.2%)	3.26	<.0001	**.005**

NOTE. The bolded values indicate statistical significance.

FDR, false-discovery rate; FODMAP, fermentable oligosaccharides, disaccharides, monosaccharides, and polyols; HC, healthy control; IBS, irritable bowel syndrome.

a Adjusted for age, sex, race, body mass index, Hospital Anxiety and Depression Scale anxiety, and FDR correction for the number of Diet Checklist items tested (N = 11).

b American diet: standard American + modified American from original Diet Checklist.

**Table 3.** Association Between Dietary Patterns and IBS Symptoms and Symptom Severity

	Standard diet (n = 206)	Nonstandard diet^a^ (n = 140)	Estimate^b^	t value	*P* value^c^	*P* value (FDR adjusted^d^)^c^

IBS-SSS, means ± SD	248.39 ± 83.85	278.95 ± 83.79	−23.20	−2.35	.019	**.042**
Abdominal pain, means ± SD	9.23 ± 4.03	9.18 ± 4.42	0.25	0.51	.612	.799
Bloating, means ± SD	10.91 ± 4.83	11.81 ± 5.21	−0.45	−0.76	.450	.566
VSI score, means ± SD	38.82 ± 15.91	45.06 ± 16.29	−4.70	−2.40	.017	.081

	Exclusion diet (n = 140)	Nonexclusion diet^e^ (n = 206)	Estimate		*P* value^f^	*P* value (FDR adjusted^d^)^f^

IBS-SSS, means ± SD	278.95 ± 83.79	248.39 ± 83.85	−23.20	−2.35	.019	**.042**
Abdominal pain, means ± SD	9.18 ± 4.42	9.23 ± 4.03	0.25	0.51	.612	.799
Bloating, means ± SD	11.81 ± 5.21	10.91 ± 4.83	−0.45	−0.76	.450	.566
VSI score, means ± SD	45.06 ± 16.29	38.82 ± 15.91	−4.70	−2.40	.017	.081

	Restrictive diet (n = 94)	Nonrestrictive diet (n = 252)	Estimate		*P* value^g^	*P* value (FDR adjusted^d^)^f^

IBS-SSS, means ± SD	291.59 ± 87.06	249.35 ± 81.57	29.33	2.69	.008	**.029**
Abdominal pain, means ± SD	9.60 ± 4.41	9.07 ± 4.10	0.07	0.13	.900	.900
Bloating, means ± SD	11.98 ± 5.66	11.01 ± 4.72	0.48	0.74	.463	.566
VSI score, means ± SD	46.31 ± 16.14	39.47 ± 16.04	5.01	2.31	.022	.081

NOTE. The bolded values indicate statistical significance.

FDR, false-discovery rate; IBS-SSS, Irritable Bowel Syndrome Severity Scoring System; SD, standard deviation; VSI, Visceral Sensitivity Index.

a Nonstandard diet equivalent to exclusion diet.

b Coefficient (*β*) for the main effect.

c *P* value comparing standard diet with nonstandard diet.

d FDR was corrected for the number of Diet Checklist items tested (N = 11).

e Nonexclusion diet equivalent to standard diet.

f *P* value comparing exclusion diet with nonexclusion diet.

g *P* value comparing restrictive diet with nonrestrictive diet.

## What You Need to Know

### Background

Irritable bowel syndrome (IBS) patients often report that symptoms worsen after meals, which can lead to avoiding certain foods. Restrictive or exclusion diets may alter the diversity and composition of the fecal microbiota.

### Findings

IBS patients on exclusion diets had worse symptoms. IBS patients on exclusion diets had greater fecal *Lachnospira* and lower *Eubacterium*. IBS patients on restrictive diets had a lower abundance of *Lactobacillus*.

### Implications for patient care

Diet influences the composition of the fecal microbiota in IBS patients, which can explain the variability of studies in IBS patients and healthy controls.

## Abbreviations used in this paper:

DHQ-II: Diet History Questionnaire-II
FDR: false-discovery rate
FODMAP: fermentable oligosaccharides, disaccharides, monosaccharides, and polyols
GI: gastrointestinal
GLM: generalized linear model
HADS: Hospital Anxiety and Depression Scale
HC: healthy control
IBS: irritable bowel syndrome
IBS-C: constipation-predominant irritable bowel syndrome
IBS-D: diarrhea-predominant irritable bowel syndrome
IBS-M: irritable bowel syndrome with mixed symptoms
IBS-SSS: Irritable Bowel Syndrome-Severity Scoring System
OR: odds ratio
OTU: operational taxonomic unit
