# Supplementary material for: Effect of Exclusion Diets on Symptom Severity and the Gut Microbiota in Patients With Irritable Bowel Syndrome
Source: Clin Gastroenterol Hepatol. Author manuscript; Available in PMC 2022 Apr 29. (PMC9054035; doi:10.1016/j.cgh.2021.05.027)
Supplement: 1 [file NIHMS1797991-supplement-1.pdf]

Supplementary Methods

Participants

IBS patients between the ages of 18 and 69 years in our research database were screened consecutively for possible inclusion in this study. The majority of patients in the research database were recruited through community advertisements for physiologic or treatment-related clinical studies by the University of California Los Angeles G. Oppenheimer Center for Neurobiology of Stress and Resilience, and a lesser proportion of patients were recruited for research studies directly through the general GI clinics at University of California Los Angeles. All participants were recruited during the time periods between July 2013 and June 2019. Patient data from previous physiologic studies was used if patients met the inclusion criteria for this study. Of note, microbiome data from only a small subset of HCs (n = 25) was published previously in studies assessing obesity, food addiction, and the brain–gut axis.[1,2] No microbiome data from IBS patients had been published previously, and no previous studies in our research database had focused on the effect of diet on the gut microbiota. This study was approved by the Institutional Review Board at the University of California Los Angeles, which allowed for the repurposing of previously collected data.

All participants underwent a medical history and physical examination. IBS participants were included in our study if they met Rome III or Rome IV criteria, depending on the year of recruitment, and were subclassified as having IBS-C, IBS-D, IBS-M, or IBS unclassified. IBS participants were excluded if they had any current organic disease that also could contribute to chronic abdominal pain (ie, inflammatory bowel disease, active peptic ulcer disease, diverticulitis, and so forth), had an overlapping dominant functional disorder such as functional dyspepsia, had any active psychiatric disease, or were on chronic opioid medications. HCs were included if they had no history of GI symptoms or an organic GI disease, and were excluded if they had any active psychiatric disease or were on chronic opioid medications. Both HCs and IBS participants who submitted stool samples for microbiota analysis were excluded if they had received antibiotics within the previous 3 months. Only a very small percentage of IBS participants (2.6%) and HCs (1.18%) reported the use of probiotics. Analyses were performed both with including and excluding these participants on probiotics, and there was no significant effect of probiotic use on our overall findings. Therefore, the participants on probiotics were included in the final analysis.

Diet Checklist

If participants did not believe that the diet they consumed most frequently was reflected by the choices of a standard American diet, modified American diet, Mediterranean diet, Paleo diet, vegan diet, vegetarian diets, gluten-free diet, dairy-free diet, or the low FODMAP diet, the option was given to select "other," and describe the components of their individual diet in regard to consumption of meat, dairy, eggs, fruits, vegetables, and grains. If a participant selected "other," their comments regarding intake of food components were reviewed individually, as was that participant's 24-hour diet diary that reflected every food component consumed in the 24-hour period before stool submission for microbiota analysis. After review of the dietary comments and 24-hour diet diaries, participants who selected "other" then were reclassified into the diet category that was most reflective of their individual diet. This then was verified against the participant's responses on the DHQ-II, a food frequency questionnaire.

If a participant selected 2 diets on the Diet Checklist, one of which fell into the standard category and one of which fell into the exclusion category, that participant ultimately was characterized as being on an exclusion diet. When available, 24-hour diet diaries were examined for participants on an exclusion diet to confirm compliance. If a participant's 24-hour diet diary was not consistent with their self-reported exclusion diet from the Diet Checklist, that participant's responses to the DHQ-II were reviewed and the diet was reclassified as appropriate.

Our institution's Diet Checklist also was validated internally against the standardized DHQ-II and each patient's 24-hour diet recall of all foods consumed in a 24-hour period before submitting a stool sample for microbiome analysis. The Food Patterns Equivalents Database from the DHQ-II, which converts foods and beverages in the Food and Nutrient Database for Dietary Studies to 37 United States Department of Agriculture Food Pattern components, was used for validation. Compliance with an exclusion diet on the Diet Checklist (dairy-free, Paleo, vegetarian, or vegan) was defined as 3 or fewer dietary indiscretions per month. This cut-off value was determined to be applicable to real-life compliance scenarios after consultation with a registered GI dietician at the University of California Los Angeles. For example, if a patient stated on their Diet Checklist that they consumed a dairy-free diet, but review of their DHQ-II showed 4 episodes of dairy intake in a 1-month period, this person was determined to not actually follow a dairy-free diet.

Using this methodology, only 2 (7.4%) subjects who indicated that they were on a Paleo diet on the Diet Checklist were nonadherent based on their DHQ-II responses, 0 (0.0%) subjects who indicated that they were on a vegan diet were nonadherent based on their DHQ-II responses, 1 subject (4.5%) who reported being on a standard vegetarian diet was nonadherent based on his or her DHQ-II responses, 0 (0.0%) subjects who indicated that they were on an ovovegetarian, lactovegetarian, or ovolactovegetarian diet were

nonadherent based on their DHQ-II responses, and 1 subject who reported consuming a dairy-free diet was nonadherent based on his or her DHQ-II responses. These subjects were reclassified into the diet category that best matched their eating patterns based on their DHQ-II responses and 24-hour diet recall. Validation was unable to be performed for the gluten-free diet and low FODMAP diet because the DHQ-II does not specifically assess consumption of gluten or distinguish between foods that are low or high in FODMAPs. For the 4 subjects who were not compliant with their indicated diet on the Diet Checklist, we then reviewed their individual Diet Checklist, DHQ responses, and 24-hour diet diary if available. Subjects' diets were reclassified into the diet category that best reflected the diet they consumed.

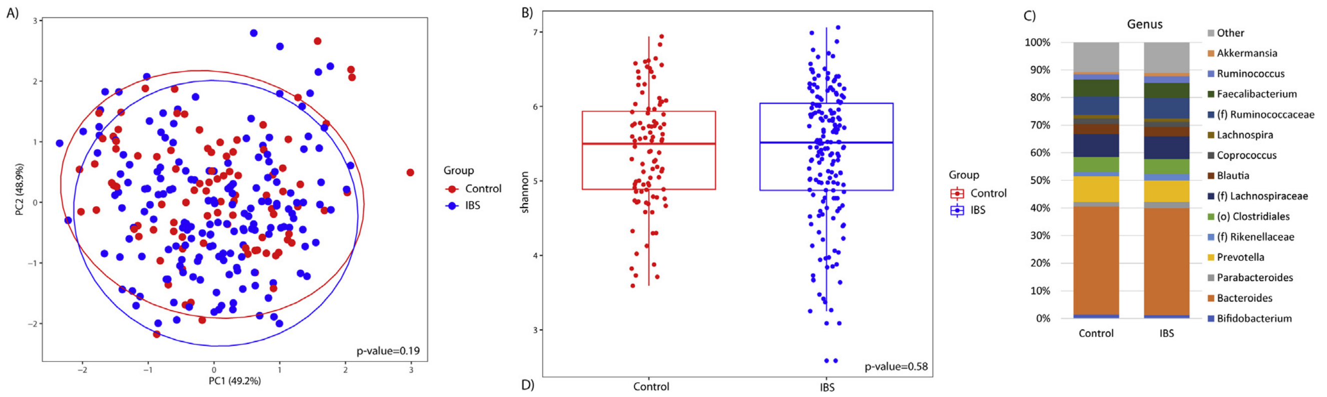

Supplementary Figure 1. (*A*) β-diversity in irritable bowel syndrome (IBS) participants vs healthy controls (HCs) irrespective of diet. (*B*) α-diversity in IBS participants compared with HCs irrespective of diet. (*C*) Taxonomic plots in IBS participants compared with HCs irrespective of diet.

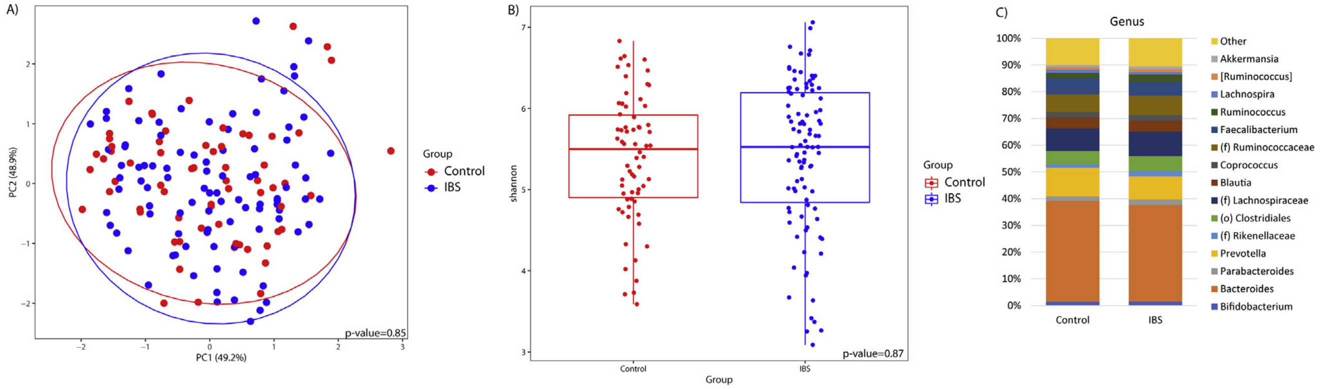

Supplementary Figure 2. (*A*) β-diversity in irritable bowel syndrome (IBS) participants on a standard diet vs not on a standard diet. (*B*) α-diversity in IBS participants on a standard diet compared with healthy controls (HCs) on a standard diet. (*C*) Taxonomic plots in IBS participants and HCs on a standard diet.

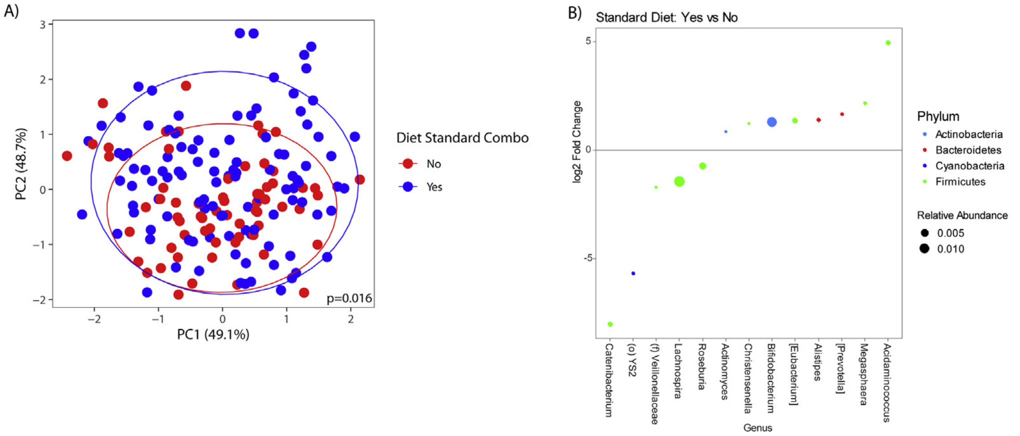

Supplementary Figure 3. (*A*) β-diversity in irritable bowel syndrome (IBS) participants on a standard diet vs not on a standard diet. (*B*) Relative fecal abundances in IBS participants on a standard diet compared with a nonstandard (exclusion) diet.

Supplementary Table 1. Diet Checklist Categories

Diet category	Components
Standard American	High consumption of processed foods, pastas, and breads<br>Meats, including red meat, fish, eggs, and dairy products consumed<br>Vegetables and fruits consumed, but not in large quantities
Modified American	High consumption of processed foods, pastas, and breads (mainly whole grain)<br>Poultry, fish, eggs, and dairy products consumed<br>Red meat consumed in limited quantities<br>Vegetables and fruits consumed, but not in large quantities
Mediterranean	High consumption of fruits, vegetables, bread and other cereals, beans, nuts, and seeds<br>Olive oil is the key monounsaturated fat source<br>Dairy products, fish, and poultry are consumed in low to moderate amounts<br>Little red meat is consumed<br>Eggs are eaten 0 to 4 times a week and wine is consumed in moderate (or low) amounts
Paleo	Consumption of basic foods such as plain meat, fish, shellfish, eggs, nuts, vegetables, fruits, berries, and mushrooms<br>Minimally processed oils, such as avocado, olive, or coconut oil, for cooking<br>Excludes dairy products, legumes, dry beans, grains, coffee, alcohol, sugar, and processed foods
Vegan	Focus is on plant-based foods<br>Includes fruits, vegetables, dried beans and peas, grains, seeds, and nuts<br>Excludes all meat and animal products
Vegetarian (6 categories)	Focus is on plant-based foods<br>Includes fruits, vegetables, dried beans and peas, grains, seeds, and nuts
Vegetarian	Excludes all meat but will allow animal-derived ingredients (ie, honey and gelatin)
Lactovegetarian	Includes plant foods plus dairy products, no eggs
Ovovegetarian	Includes plant foods plus eggs, no dairy
Lacto-ovovegetarian	Includes both dairy products and eggs
Pescatarian	Includes fruits, vegetables, dried beans and peas, grains, seeds, and nuts<br>Excludes all meat except fish
Raw vegan/raw food	Unprocessed vegan foods that have not been heated to higher than 115°F (46°C)
Gluten-free	Diet includes most foods but avoids the protein gluten, which is found in wheat, barley, and rye
Dairy-free	Diet includes most foods but avoids dairy
Low FODMAP	Diet limits foods high in sugar and carbohydrates (fructose, lactose, fructans, galactans, and polyols)

FODMAP, fermentable oligo-, di-, monosaccharides, and polyols.

Supplementary Table 2. Components of the IBS-SSS and the Relationship With Exclusion and Restrictive Diets

	Exclusion diet	Nonexclusion diet	*P* value	Restrictive diet	Nonrestrictive diet	*P* value
Question 1a (abdominal pain, yes, %)	96	94	.537	94	95	.928
Question 1b (severity of abdominal pain [0–100]; means ± SD)	47.18 ± 20.39	44.42 ± 20.02	.230	48.53 ± 21.92	44.45 ± 20.39	.108
Question 1c (number of days of abdominal pain in past 10 days; means ± SD)	5.55 ± 2.77	4.46 ± 2.60	**<.0001**	5.88 ± 2.87	4.54 ± 2.77	**<.0001**
Question 2a (abdominal distention, yes, %)	89	82	.069	92	82	**.021**
Question 2b (severity of abdominal distention [0–100]; means ± SD)	69.15 ± 24.06	68.86 ± 23.1	.054	72.05 ± 24.62	67.84 ± 24.06	.088
Question 3 (bowel habit satisfaction [0–100]; means ± SD)	69.15 ± 24.06	68.86 ± 23.1	.914	72.05 ± 24.62	67.84 ± 24.06	.143
Question 4 (IBS affecting quality of life [0–100]; means ± SD)	64.19 ± 20.03	56.99 ± 22.34	**<.001**	67.74 ± 18.87	57.00 ± 20.03	**<.001**

NOTE. The bolded values indicate statistical significance.
IBS-SSS, Irritable Bowel Syndrome Severity Scoring System; SD, standard deviation.

Supplementary Table 3. Diet Categories and Bowel Habit Subtypes in IBS Participants

Diet	IBS-C, n	IBS-D, n	IBS-U, n	IBS-M, n	χ^2 value	*P* value	FDR-adjusted *P* value
Standard diet	64	77	11	52	6.14	.105	.262
American	65	89	12	48	2.38	.498	.685
Mediterranean	16	7	3	14	10.90	.012	.132
Exclusion diet	40	62	14	24	6.14	.105	.262
Vegan	1	6	1	1	3.36	.340	.534
Vegetarian (all subtypes)	12	19	3	9	0.28	.963	.981
Paleo	9	10	2	6	0.18	.981	.981
Low FODMAP	6	5	2	5	1.78	.620	.758
Gluten-free	17	19	7	9	4.14	.247	.453
Dairy-free	15	32	8	8	9.15	.027	.148
Restrictive diet	28	40	11	15	5.86	.119	.262
Low FODMAP	6	5	2	5	1.78	.620	.758
Gluten-free	17	19	7	9	4.14	.247	.453
Dairy-free	15	32	8	8	9.15	.027	.148

FDR, false-discovery rate; FODMAP, fermentable oligo-, di-, monosaccharides, and polyols; IBS-C, constipation-predominant irritable bowel syndrome; IBS-D, diarrhea-predominant irritable bowel syndrome; IBS-M, irritable bowel syndrome with mixed symptoms; IBS-U, irritable bowel syndrome unclassified.

Supplementary Table 4. Association Between DHQ-II Food Variables and IBS

Food variable	HC, means (SD)	IBS, means (SD)	Z-value	OR (95% CI), IBS vs HCs	FDR adjusted *P* value
Energy, *kcal/d*	1995.62 (1589.95)	1853.51 (926.57)	-2.16	1.00 (0.99–1.00)	.078
Total protein, *g/d*	84.45 (82.72)	76.29 (44.56)	-2.35	0.99 (0.99–01.00)	.052
Animal protein, *g/d*	52.58 (63.42)	47.13 (34.89)	-1.97	0.99 (0.98–1.00)	.083
Vegetable protein, *g/d*	31.85 (24.05)	29.13 (16.98)	-2.80	0.97 (0.96–0.99)	.026
Total fat, *g/d*	79.49 (70.65)	74.29 (42.44)	-1.80	0.99 (0.99–1.00)	.113
Total saturated fat, *g/d*	24.43 (22.91)	22.42 (13.92)	-1.92	0.98 (0.96–1.00)	.089
Total monounsaturated fat, *g/d*	30.46 (27.23)	28.93 (17.44)	-1.63	0.99 (0.97–1.00)	.145
Total polyunsaturated fat, *g/d*	17.16 (15.17)	16.07 (9.55)	-1.70	0.98 (0.95–1.00)	.132
Total sugars, *g/d*	100.01 (71.45)	89.47 (52.45)	-2.61	0.99 (0.98–0.99)	.038
Fructose, *g/d*	28.05 (30.07)	25.00 (20.86)	-1.61	0.99 (0.98–1.00)	.145
Galactose, *g/d*	0.59 (0.66)	0.45 (0.38)	-3.63	0.17 (0.06–0.39)	**.005**
Glucose, *g/d*	26.23 (22.89)	23.90 (16.41)	-2.00	0.98 (0.96–1.00)	.083
Lactose, *g/d*	0.59 (0.66)	0.45 (0.38)	-3.63	0.17 (0.06–0.39)	**.005**
Maltose, *g/d*	3.48 (3.21)	3.59 (3.85)	-2.08	0.90 (0.82–1.00)	.078
Sucrose, *g/d*	35.36 (29.90)	31.72 (20.42)	-2.10	0.98 (0.97–1.00)	.078
Starch, *g/d*	96.34 (83.94)	89.78 (55.85)	-2.05	0.99 (0.99–1.00)	.078
Total dietary fiber, *g/d*	25.54 (19.67)	22.85 (13.98)	-3.06	0.96 (0.94–0.99)	**.017**
Soluble fiber, *g/d*	7.78 (5.05)	6.99 (4.35)	-3.50	0.86 (0.79–0.93)	**.005**
Insoluble fiber, *g/d*	17.72 (15.23)	15.80 (10.38)	-2.83	0.96 (0.93–0.98)	**.026**
Total grains, *g/d*	4.93 (4.75)	4.80 (3.54)	-0.06	0.99 (0.58–1.70)	.954
Whole grains, *g/d*	0.60 (0.49)	0.70 (0.70)	-1.58	0.93 (0.84–1.01)	.147
Refined grains, *g/d*	4.34 (4.47)	4.10 (3.14)	-1.50	0.94 (0.86–1.02)	.166
Carbohydrates, *g/d*	238.94 (175.10)	218.89 (109.67)	-2.49	1.00 (0.99–1.00)	**.044**
Total meat, *oz/d*	1.470 (2.88)	1.19 (1.87)	-1.39	0.91 (0.77–1.03)	.198
Cured meat, *oz/d*	0.69 (1.41)	0.52 (0.77)	-1.21	0.84 (0.61–1.10)	.259
Organ meat, *oz/d*	0.04 (0.22)	0.01 (0.02)	-0.89	0.07 (NA to 1.16)	.417
Total vegetables, *cup equivalents/d*	3.06 (3.26)	2.52 (2.02)	-2.58	0.83 (0.71–0.95)	**.038**
Total fruits, *cup equivalents/d*	1.73 (2.02)	1.69 (1.82)	-0.73	0.93 (0.75–1.12)	.495
Total dairy, *cup equivalents/d*	1.34 (1.26)	1.17 (1.01)	-2.44	0.65 (0.46–0.90)	**.045**
Alcohol, *g/d*	5.58 (8.17)	6.90 (10.78)	0.37	1.01 (0.97–1.04)	.734

NOTE. The bolded values indicate statistical significance.
DHQ-II, Diet History Questionnaire-II; FDR, false-discovery rate; HC, healthy control; IBS, irritable bowel syndrome; OR, odds ratio; SD, standard deviation.

Supplementary Table 5. Differences in the Fecal Microbiota Observed Between HCs and IBS, Adjusted for Race

Family	Genus	Base mean	Relative abundance	Log_2 fold change	lfcSE	Stat	*P* value	Adjusted *P* value	Q value
Rikenellaceae		2682.122	0.020	0.960	0.263	3.655	.000	.013	0.010
Porphyromonadaceae	*Parabacteroides*	2934.118	0.0221	0.708	0.236	3.001	.003	.0446	0.0335

HC, healthy control; IBS, irritable bowel syndrome; lfcSE, standard error of the log2FoldChange estimate; Stat, Wald statistic.

Supplementary Table 6. Differences in the Fecal Microbiota Observed Between HCs and IBS, Adjusted for Diet and Race

Family	Genus	Base mean	Relative abundance	Log_2 fold change	lfcSE	Stat	*P* value	Adjusted *P* value	Q value
Rikenellaceae		2682.122	0.020	0.955	0.268	3.566	.000	.018	0.014

HC, healthy control; IBS, irritable bowel syndrome; lfcSE, standard error of the log2FoldChange estimate; Stat, Wald statistic.

Supplementary Table 7. Summary of Previous Evidence of Dietary-Induced Changes in the Genera Pertinent to this Study

Genus	Details	Pertinent previous dietary studies in IBS patients	Pertinent previous dietary studies in non-IBS patients	Findings in this study
Bifidobacterium	Gram-positive, nonmotile anaerobic bacteria<br>Some bifidobacterial are used as probiotics given positive health benefits	IBS patients on a habitual diet had a greater fecal concentration of *Bifidobacterium* compared with patients on the low FODMAP diet<br>Likely related to a greater abundance of fructans<br>A paradoxic increase in IBS symptoms was seen with the habitual diet[3]<br>IBS patients on a traditional diet had greater fecal Bifidobacterium compared with patients on a low FODMAP diet[4]	*Bifidobacterium*-containing probiotics associated with a reduction in IBS symptoms compared with probiotics containing *Lactobacillus* alone[5]	IBS participants on a standard diet had a significantly greater fecal abundance of *Bifidobacterium* and lesser IBS symptom severity compared with IBS participants not on a standard diet
Prevotella	Gram-negative, predominantly commensal, rod-shaped bacteria	–	*Prevotella* is associated with diets high in carbohydrates and simple sugars[6]<br>*Prevotella* ferments dietary fiber into short-chain fatty acids[7]	IBS participants on a standard diet had a significantly greater fecal abundance of *Prevotella* compared with IBS participants not on a standard diet
Lachnospira	Gram-positive, non–spore-forming, anaerobic, rod-shaped bacteria, involved in the fermentation of pectin, a complex fiber and heteropolysaccharide found in the cell walls of certain fruits and vegetables<br>*Lachnospira* also are known to be short-chain fatty acid producers, which can have positive effects on immune function, intestinal barrier integrity, mucus production, and overall gut health	–	Vegetable intake was associated positively with an increased relative abundance of *Lachnospira*[8]<br>Vegetable-based diet associated with *Lachnospira*[9]	IBS participants on an exclusion diet had a significantly greater abundance of *Lachnospira* compared with those not on an exclusion diet

Supplementary Table 7. Continued

Genus	Details	Pertinent previous dietary studies in IBS patients	Pertinent previous dietary studies in non-IBS patients	Findings in this study
Eubacterium	Heterogenous group of Gram-positive, non–spore-forming, butyrate-producing, anaerobic bacilli	–	Eight-week trial of a gluten-free diet in healthy individuals associated with a decreased abundance of *Eubacterium hallii*, which may have been partially driven by changes in dietary fiber[10]	IBS participants on an exclusion diet had a lower fecal abundance of *Eubacterium* compared with those not on an exclusion diet
Lactobacillus	Gram-positive, anaerobic or microaerophilic, rod-shaped bacteria that convert sugars to lactic acid and are considered beneficial to human health	–	A gluten-free diet over a 1-month period in healthy individuals was associated with a decreased abundance of Lactobacillus[11]<br>The ratio of *Lactobacillus:Bifidobacterium* to *Bacteroides-Escherichia coli* was reduced significantly in celiac patients on a gluten-free diet (with either active or inactive disease) compared with controls[12]	IBS participants on a restrictive diet had a lower fecal abundance of Lactobacillus compared with IBS participants not on a restrictive diet

FODMAP, fermentable oligosaccharides, disaccharides, monosaccharides, and polyols; IBS, irritable bowel syndrome.
